# Evaluation of Functional Activity of Ciliated Epithelium in Patients with Primary Ciliary Dyskinesia

**DOI:** 10.3390/diagnostics16213521

**Published:** 2026-10-27

**Authors:** Tatiana Kyian, Anna Demchenko, Olga Solovova, Maxim Tarasov, Svetlana Smirnikhina, Aleksandra Nazarova, Elizaveta Bragina, Oksana Ryzhkova, Elena Kondratyeva, Sergey Kutsev

**Affiliations:** 1 Federal State Budgetary Scientific Institution “ Research Centre for Medical Genetics” (RCMG), 1 Moskvorechye St., 115522 Moscow, Russia; 2 A.N. Belozersky Institute of Physico-Chemical Biology, Lomonosov Moscow State University, 119992 Moscow, Russia

**Keywords:** primary ciliary dyskinesia, ciliogenesis, ciliary beat frequency, ALI culture *in vitro*, biopsy specimens, genes

## Abstract

Primary ciliary dyskinesia (PCD) is a heterogeneous recessive disorder of cilia that leads to respiratory tract disorders and organ laterality defects. **Objectives:** This study was designed to diagnose PCD in patients using various methods: genetic, immunofluorescence, transmission electron microscopy (TEM), and assessment of ciliary beat frequency (CBF) in ALI cultures. **Methods:** The study included (*n* = 35) patients with primary ciliary dyskinesia and (*n* = 10) healthy controls (HC). The diagnosis of PCD was established using molecular genetic analysis: WES, TEM, immunofluorescence staining of ALI-culture, and high-speed videomicroscopy. Ciliogenesis of ciliated epithelial cells was performed in patients with PCD and HC to improve the diagnosis of PCD. **Results:** Genetic sequencing was conducted for all patients and variants in 17 genes were found. All variants were known. A comparison of CBF assessment in PCD patients and HC using high-speed videomicroscopy showed that the CBF in PCD patient nasal cells was significantly reduced (*p* < 0.0001), with sensitivity of 91% and specificity of 89.2%. CBF was independent of age and showed no statistical difference between adults and children. **Conclusions:** The diagnosis of PCD was confirmed in all patients using various diagnostic methods, including the assessment of CBF of ALI culture. The creation of databases of ALI cultures, combined with genotypic and structural abnormalities, is necessary both for diagnosis and, in the future, for use as models in the development of targeted therapies and for assessing their effectiveness.

## 1. Introduction

PCD is a ciliopathy characterized by impaired movement of the cilia in the airways and sinuses, which hinders normal mucociliary clearance and causes a range of symptoms [1].

Primary cultures of human respiratory epithelial cells are an indispensable tool in studies of the pathophysiology of respiratory tract diseases. One of the applications of respiratory epithelial cell cultures is differentiation in the ciliary direction, which is carried out using the air–liquid interface (ALI) culturing method, with subsequent functional analysis of the obtained cilia [2,3]. This method has an important application in the diagnosis of PCD [4]. PCD is a heterogeneous disease in which variants arise in genes encoding axonemal structure proteins, regulatory complexes, ciliary body assembly, or ciliary transport proteins [5]. Abnormalities in cilia function depend on the underlying defect, which can lead to the development of static (immobile) cilia, with markedly reduced beat amplitude, as well as cilia that rotate or are hyperkinetic [6]. The clinical signs of PCD usually begin at birth, with respiratory distress syndrome or congenital pneumonia followed by a wet cough in early childhood and the development of bronchiectasis, chronic sinusitis and otitis media [7].

However, PCD is not only a respiratory disease. Male infertility is a common related phenomenon, because sperm flagella are special motile cilia that can also exhibit defective motility [8]. Motile cilia line the apical surface of epithelial cells along the upper and lower respiratory tracts, male and female reproductive tracts (fallopian tubes and efferent tubules), and ependymocytes of the ventricles of the brain. There are usually hundreds of motile cilia per epithelial cell. The main function of cilia is to vector fluid and mucus parallel to the cell surface in a coordinated manner. Spermatozoa also have a single flagellum, structurally similar to motile cilia, which is responsible for promoting sperm [9,10].

Specialized centers use cultures of ciliated cells obtained in vitro for differential diagnosis with secondary ciliary dyskinesia; this may arise due to chronic inflammation, infection and the prolonged use of nasal decongestants, which may complicate the interpretation of the diagnosis [11]. ALI culture may also decrease the need for repeat ciliary biopsy [12]. Moreover, immunofluorescence analysis of PCD marker proteins is performed on ALI cultures, which contributes to the characterization of various PCD phenotypes [13].

In this study, ciliogenesis of ciliated epithelial cells was performed in patients with primary ciliary dyskinesia and healthy controls (HC) to improve the diagnosis of PCD. The high-speed videomicroscopy (HSVM) analysis of CBF in the obtained ALI cultures and ex vivo was carried out in patients with PCD in comparison with HC. Immunofluorescence of ALI cultures, transmission electron microscopy (TEM) and molecular genetic studies were performed to confirm the diagnosis.

The aim of the study was to diagnose PCD in patients using various methods: genetic, immunofluorescence, transmission electron microscopy (TEM), and the assessment of ciliary beat frequency (CBF) in ALI cultures.

## 2. Materials and Methods

The study was approved by the Ethics Committee of the Research Centre for Medical Genetics № 4 of 19.04.21 (Moscow, Russia) and conducted in accordance with the provisions of the Declaration of Helsinki of 1975. Patients and healthy controls signed informed written consent forms as anonymous participants in the study and donors of biological materials—nasal epithelium cells obtained via the brush biopsy method from patients and healthy controls [11]. A disposable cytology brush, model №BC-202D-2010 by Olympus, channel size 2.00, 3.00, 5.00 mm, was used. Informed consent was obtained for diagnostic tests, after explanations were provided of all procedures to parents and subjects. Informed consent was obtained from the HCs.

The study included (*n* = 35) patients with suspected PCD from Moscow and the Moscow region: 17 (49%) were female and 18 (51%) were male, with a median age of 15 |2–44|. Among the *n* = 10 HC, 4 (40%) were female and 6 (60%) were male with median age 24 |2–61|.

Childhood was defined as age from 0 to 18 years old.

Reviewed clinical data, PICADAR score, age, sex, genotype and respiratory microbiology were collected from electronic medical records, before being anonymized and transcribed.

In 35 patients with PCD and 10 HCs, the nasal epithelium was obtained via brush biopsy. At the time of admission, 3 brush biopsies at the same visit from the different sites (nostril) were performed on each patient and HC for high-speed video microscopy in vitro, TEM and culturing.

**Inclusion criteria for PCD patients:**

1. Anamnesis of PCD.
2. Results of diagnostic tests: decreased lung function in spirometry, isolation of pathological microflora, bronchiectasis according to lung CT scan, PICADAR [14] score > 5 points. Chronic rhinosinusitis without nasal polyps and with nasal polyps (CRS) and/or otitis media/hearing loss, according to European and Russian clinical guidelines, 2025 [15,16], and informed consent.

**Exclusion criteria:**

1. Lack of informed consent of patients and/or their parents.
2. Exacerbation of chronic rhinosinusitis.
3. Presence of viral or bacterial infection for less than two weeks prior to the study.

**Criteria for inclusion in the group of healthy children and adults**: no signs of acute disease at the time of examination, no signs of PCD, and informed consent.

A disposable cytological brush BC-202D-5010 with a length of 1150 mm, a diameter of 5.0 mm for adults and 2.00, 3.00 and 5.00 mm for children (Olympus Medical Systems Corp. Tokyo, Japan) was used.

### 2.1. Obtaining Primary Cultures of Human Nasal Epithelial Cells

**Figure 1:**
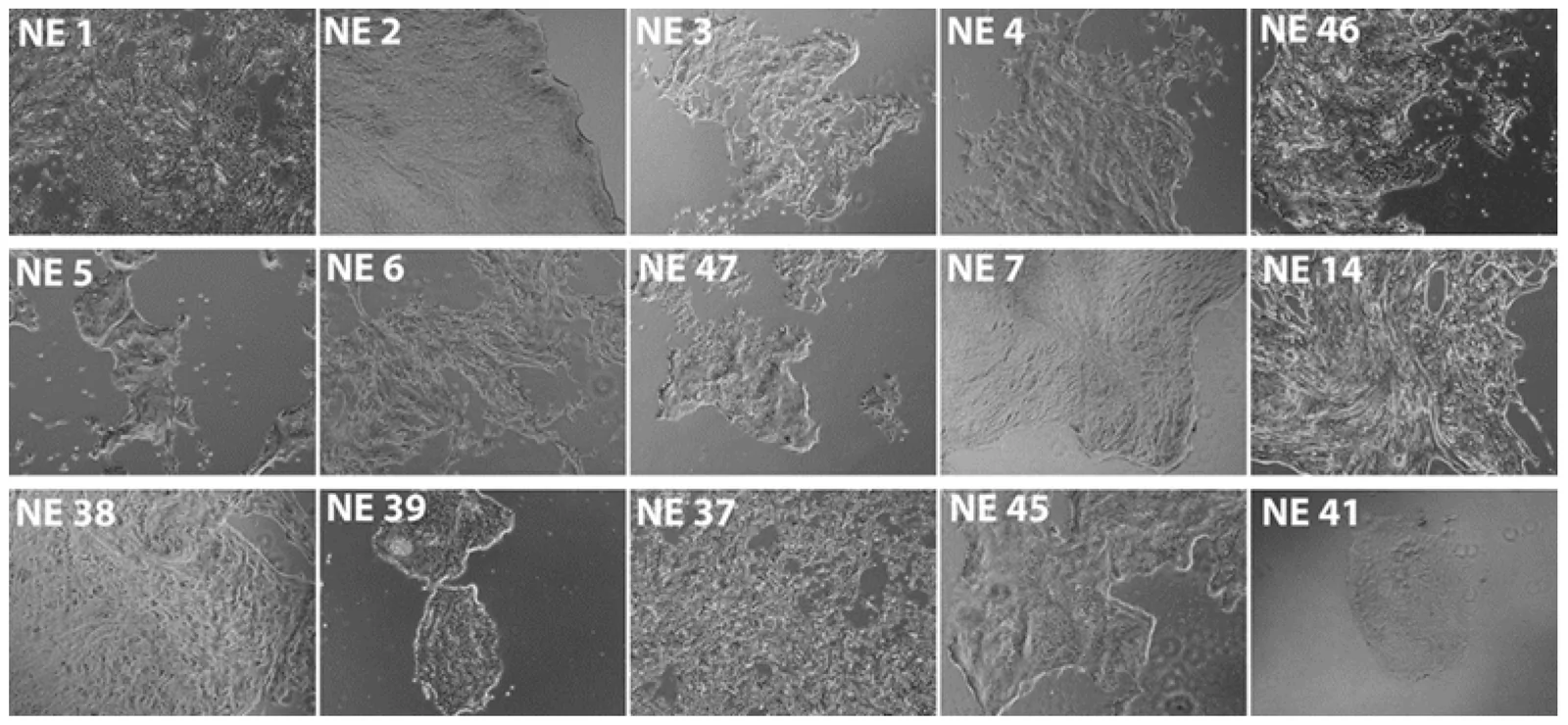
Phase-contrast image of the nucleus cells of the nasal cell at 0 passages. Increase: 100 times. NE—nasal epithelium.

To obtain primary cultures of nasal epithelial cells from patients with PCD and (*n* = 10) healthy controls (HC), the biomaterial was placed in a sterile tube containing a transport medium consisting of DMEM (PanEco, Moscow Region, Russia), 10 µg/mL of fungin (InvivoGen, France), 100 u/mL of penicillin and 100 µg/mL of streptomycin (PanEco, Russia). If necessary, transportation and storage of the obtained biomaterial were carried out in a medium for transportation at a temperature of 4 °C. The resulting nasal biopsies were centrifuged for 5 min at 1500 *g* and, under sterile conditions provided by laminar flow, placed on a culture plastic pre-coated with Matrigel (Corning, New York state, USA) in an environment for epithelial basal cells. The medium for epithelial basal cells consisted of PneumaCult™-Ex Plus Medium (StemCell Technologies, Vancouver, BC, Canada), 1 µM A83-01 (Tocris, Bristol, UK), 1 µM DMH1 (Tocris, Bristol, UK), 1 µM hydrocortisone (Sigma Aldrich, St. Louis, Missouri, USA), 50 u/mL penicillin and 50 µg/mL streptomycin (PanEco, Moscow Region, Russia). The cells were cultured in a CO_2_ incubator at a temperature of 37 °C with 5% CO_2_. Figure 1 shows a phase-contrast image of the cells isolated during brush biopsy at the zero passage. The nasal epithelial cells were passaged every 4–5 days using a 0.25% Trypsin solution (PanEco, Moscow Region, Russia). In 1–2 passages, cryopreservation of cells was performed in an amount of 1 mL at a concentration of 1 × 10^6^ cells/mL. The cells were counted using an automatic Countess II cell counter (Thermo Fisher Scientific, Waltham, MA, USA). Cryopreservation was performed using 90% bovine fetal serum (Thermo Fisher Scientific, Waltham, MA, USA) and 10% dimethyl sulfoxide (PanEco, Russia). Biobanking of the obtained cells was carried out.

### 2.2. Ciliogenesis of Human Nasal Epithelial Cells In Vitro

Ciliogenesis of 45 cultures of nasal epithelial cells obtained from 10 healthy donors and 35 patients with PCD (Table 1) was performed via ALI culturing [3]. For this purpose, culture inserts with a pore size of 0.4 μm and a diameter of 6.5 mm (StemCell Technologies, Canada) were coated with a solution of 0.3 mg/mL of type I collagen (Gibco, USA) in 20 mM acetic acid. Then, the inserts were washed three times from the residual collagen solution using DPBS solution (PanEco, Russia). Nasal epithelial cells were passaged onto the inserts at an amount of 30 × 10^3^ cells/insert for 1–2 passages. Cell counting was performed on an automatic Countess II cell counter. Epithelial basal cell medium was added 500 µL under the insert and 200 µL over the insert. When the cells reached a monolayer, the medium above the insert was completely removed and the medium below the insert was replaced with the ALI culture medium. The ALI culture medium consisted of PneumaCult™-ALI Medium (StemCell Technologies, Canada), 4 μg/mL heparin (StemCell Technologies, Canada), 9.6 μg/mL hydrocortisone (StemCell Technologies, Canada), 50 units/mL penicillin and 50 μg/mL streptomycin. ALI culturing was performed for 24 days with medium changes every 48 h (Figure 1).

### 2.3. High-Speed Videomicroscopy (HSVM) Analysis

Cultured cells were placed on glass slides in a prewarmed medium. For video microscopy, a Vert A1 inverted microscope (Zeiss, Shanghai, China) with the lens at 100× with a cumulative increase of 1000× and a removable 1.5 MP ½.9 Sony Exmor CMOS Sensor (E3ISPM01500KPA, Sony, Tokyo, Japan) was used, with a rate of 100–120 frames per second (fps) for a duration of one minute, and intervals of 10 s.

Video images were analyzed using the “Program for determining the beating frequency of the ciliated epithelium in primary ciliary dyskinesia” (PCD High-Speed Video Microscopy Analysis (PCD HSVMA)), developed in the Russian Federation, registration number No2023687245 [17]. The efficiency of the program was checked by analyzing native preparations and ALI cultures of the ciliated epithelium.

### 2.4. Transmission Electron Microscopy

To assess ultrastructural changes in the ciliated epithelium, brush biopsies were fixed in 2.5% glutaraldehyde solution at 0.1 M cacodylate buffer for 24 h, fixed with 1% osmium tetrachloride solution on distilled H_2_O for 2 h, dehydrated in a series of alcohols of ascending concentrations (50%—15 min, 70%—12 h, 96%—2 shifts for 60 min) and in acetone (30 min). After dehydration, liquid epoxy resin (Epon 812, DDSA, MNA and DMP, mixed according to the manufacturer’s prescription) was impregnated in mixtures with acetone (1:2—1 h, 1:1—1 h, 2:1—12 h) and poured into liquid epoxy resin with a catalyst. Polymerization took place for 24 h at 37 °C and 24 h at 60 °C. A microscope (JEM-1011 (JEOL, Akishima, Japan)) equipped with an Orius SC1000 W camera (Gatan Inc., Pleasanton, CA, USA) at 80 kV was used [12].

According to final guideline (a), a definition of Class 1 cilia defects diagnostic for PCD, (b) identifies Class 2 defects that can indicate a diagnosis of PCD in combination with other supporting evidence [18].

### 2.5. Immunofluorescence Staining of ALI Cultures

Immunofluorescence staining of the ALI culture of ciliary cells took place on the 24th day of ciliary differentiation. β-tubulin positively stained the ciliary structures, indicating that ciliary cells are formed in the culture upon completion of ciliogenesis in vitro. We produced fluorescent images with β-tubulin and markers of ciliated cell structural proteins (DNAH5, DNAH11, HYDIN, CFAP300) in the genes of which a pathogenic variant is identified in each specific patient.

### 2.6. Standard Semen Examination

Semen samples were collected via masturbation after 3–5 days of sexual abstinence. Patients underwent a standard semen analysis according to the recommendations of the WHO guidelines [19]. Analyzed semen parameters included ejaculate volume, viscosity and pH, concentration and total sperm count, motility, vitality and morphology. Sperm vitality (%), motility (%) and morphology (%) and sperm count were determined via light microscopy using a Nikon Ci microscope (Nikon Corp., Tokyo, Japan). Reference values of semen parameters are given according to WHO guidelines [19].

### 2.7. Genetics

For molecular genetic investigations, blood samples were obtained from both affected patients and healthy family members. Exome sequencing was conducted for all patients involved in the study. Target enrichment was achieved using the Illumina TruSeq^®^ ExomeKit (Illumina, San Diego, CA, USA), along with custom oligonucleotides (IDT xGen Exome Research Panel v1.0), which encompassed the coding regions of over 20,000 protein-coding genes. Paired-end sequencing (2 × 150 bp) was performed on an Illumina NextSeq 500. The sequencing data were analyzed using Illumina’s Basespace software (Enrichment 3.1.0). Variants were filtered based on their frequency, considering only those with a frequency of less than 1% in the Genome Aggregation Database (gnomAD v.2.1.1) and focusing on coding region sequence effects such as missense, nonsense, coding indels, and splice site alterations. The clinical relevance of the variants was assessed according to the ACMG criteria for variant interpretation. To confirm the pathogenic variant in the affected patient and their family members, Sanger sequencing was conducted using the Applied Biosystems 3130 xl Genetic Analyzer (HITACHI, Applied Biosystems Group of The Applera Corporation Japan, Waltham, MA, USA) [20,21].

### 2.8. Statistical Analysis

Statistical analysis was performed in GraphPad Prism version 8 using one-way ANOVA. Depending on the type of distribution, measures of central tendency and dispersion for quantitative data were median (Me) with description of interquartile intervals [UQ-LQ]. Diagnostic performance of CBF was assessed by ROC analysis. Several CBF recordings were obtained from each ALI culture; the primary analysis was therefore performed at the level of the subject, using the median CBF across all recordings from that subject. An analysis at the level of individual recordings is presented as supplementary, with confidence intervals obtained by bootstrap resampling of subjects (4000 replicates) to account for the non-independence of repeated recordings. The area under the curve (AUC) is reported with its 95% confidence interval. Cut-offs were evaluated using the rule “PCD if CBF < cut-off”. For each cut-off, a 2 × 2 classification table was constructed and sensitivity, specificity, positive and negative predictive values, accuracy, positive and negative likelihood ratios and the Youden index (J = sensitivity + specificity − 1) were calculated, with 95% confidence intervals for all proportions computed via the Wilson score method. Three cut-offs are reported: the value maximizing the Youden index, the highest cut-off achieving 100% specificity and the lowest cut-off achieving 100% sensitivity. Because predictive values and accuracy depend on the proportion of affected individuals in the sample tested, predictive values were additionally recalculated using Bayes’ theorem for pre-test probabilities of 10%, 20% and 30%. Recordings in which no ciliary motion was detected were included at the software’s lower limit of detection.

## 3. Results

According to anamnestic data analysis, clinical examination, instrumental examination, and molecular genetic study, complete PCD with situs inversus totalis was detected in 14 (40.0%) of the examined patients. CRSwNP or CRSsNP was diagnosed in 35 (100%) patients. Conductive hearing loss was diagnosed in nine (13.3%) patients. A CT scan of chest organs was performed in 100% of subjects, and bronchiectasis was detected in 22 (63.0%) patients. Chronic *Pseudomonas aeruginosa* infection was noted in 16 (40%) patients. According to the sperm analysis, patients with variants in the genes *DNAH17*, *CCDC39*, and *OFD1* exhibit total asthenoteratozoospermia. Notably, characteristic PCD-related ultrastructural axonemal defects were identified in the patient with DNAH17 variants, which were previously associated solely with isolated infertility [22]. This finding potentially expands the phenotypic spectrum of *DNAH17* pathogenic variants and suggests a possible overlap between isolated male infertility and systemic ciliopathy syndromes. Molecular genetic diagnosis was established in all patients. Variants in 16 genes with autosomal recessive type of inheritance were found in this cohort: *DNAAF11*, *DNAH5*, *HYDIN*, *OFD1*, *DNAH11*, *DNAH9*, *DNAL1*, *DNAAF1*, *DNAH14*, *CFAP300*, *FSIP2*, *CCDC40*, *CCDC103*, *DNAH17*, *DNAAF4*, *CCDC39* and 1 X-linked gene, *OFD1* (Table 1).

All patients underwent TEM studies. According to the international consensus guideline for reporting transmission electron microscopy: class 1 defects were detected in 20 (57%) patients, class 2 defects were identified in 7 (20%) normal ultrastructure was found in 2 (6%), and no samples were found in 6 (17%) patients (Table 1) [18,22,23].

**Figure 2:**
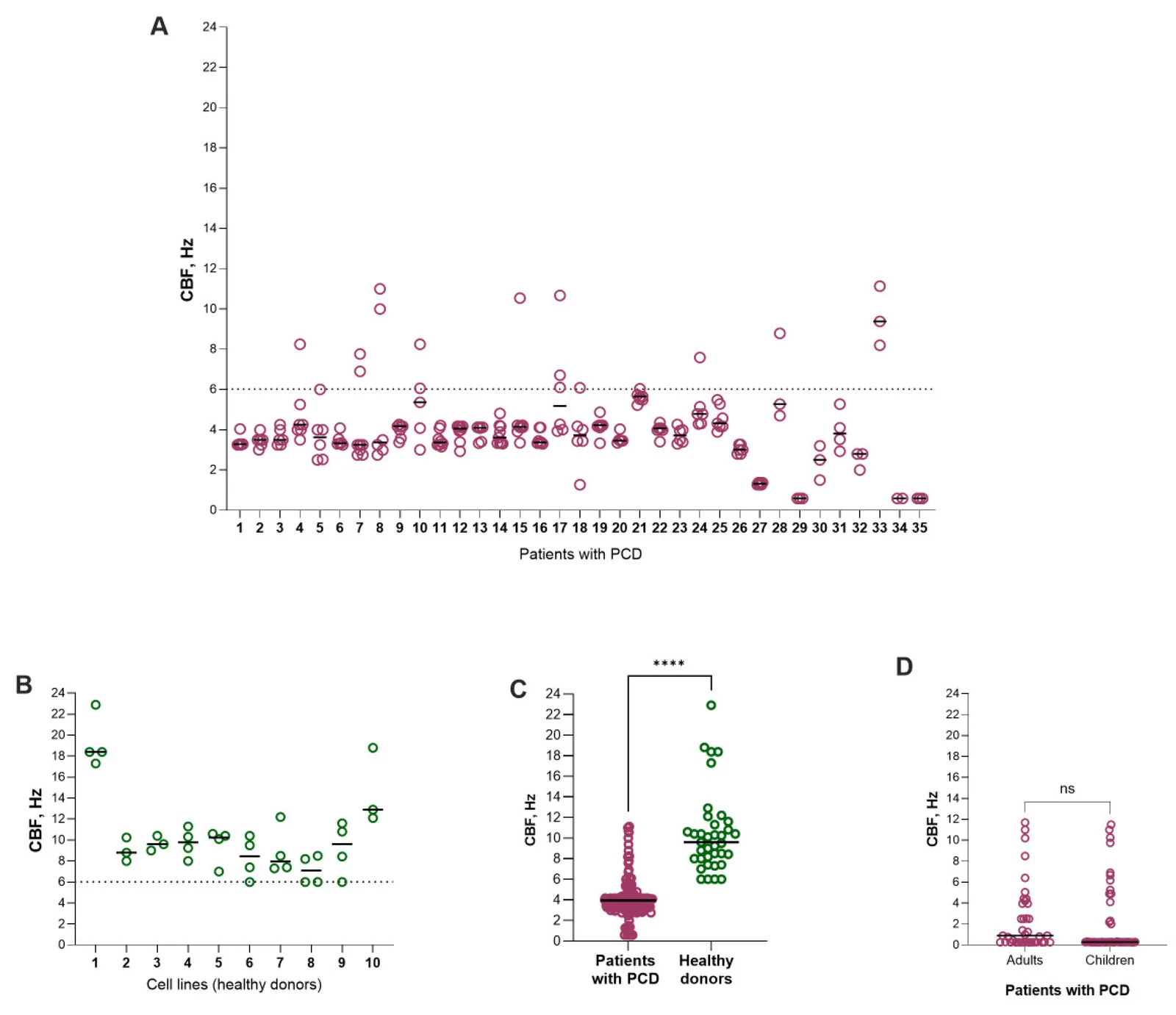
(**A**)—patients with PCD, (**B**)—healthy donors, (**C**)—comparison of the CBF (ciliary beat frequency) of patients with PCD and healthy donors, (**D**)—difference in CBF between children and adults with PCD. ****, ns.

**Figure 3:**
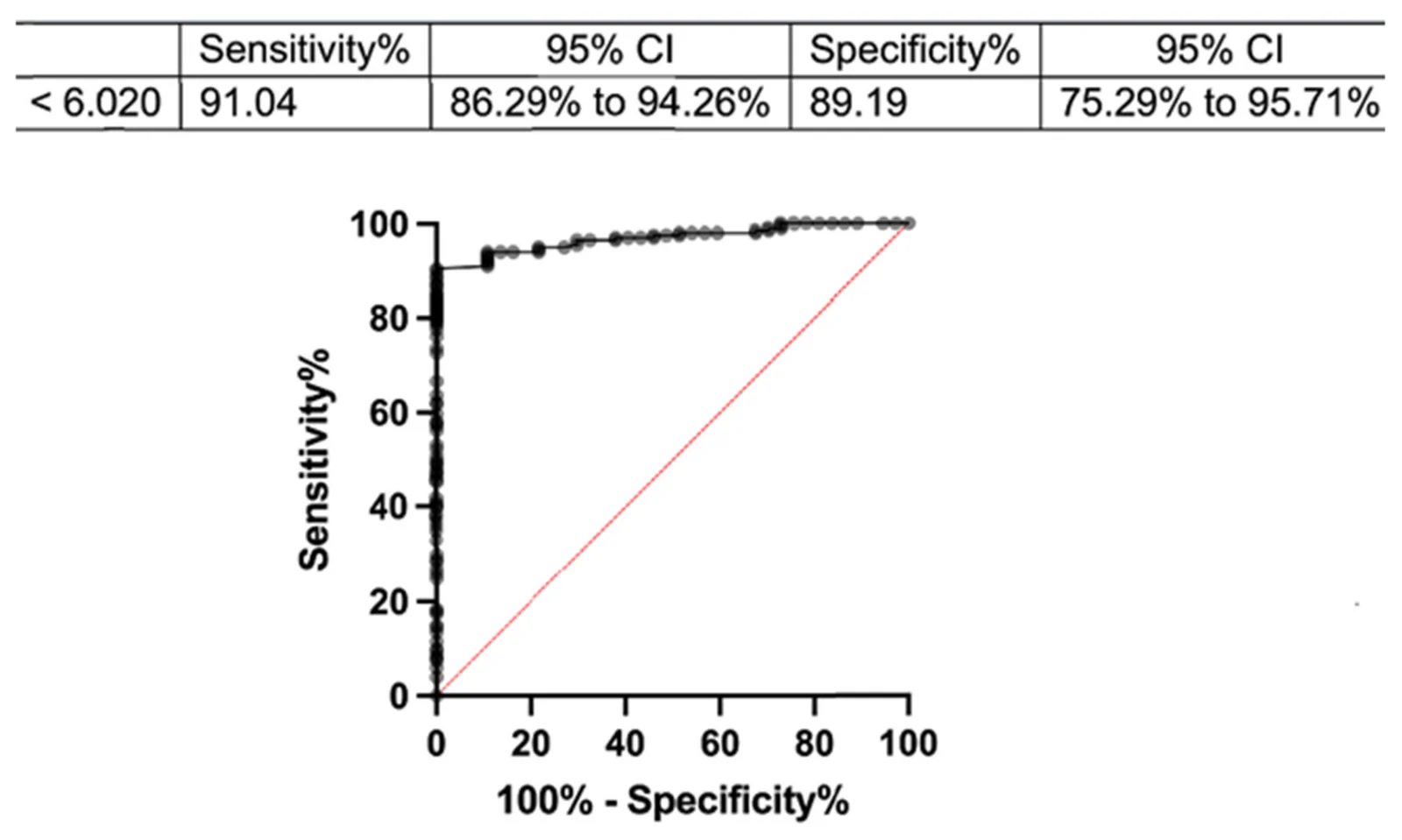
ROC analysis. A threshold was found for patients with PCD, CBF less than 6.020 Hz. ROC—receiver operating characteristic; CBF—ciliary beat frequency.

A comparison between patients and healthy controls in ALI culture using the PCD HSVMA program showed that CBF in controls was significantly higher than in patients: median 9.61 Hz [8.54–10.13] versus 3.62 Hz [3.31–4.20] (*p* < 0.0001, Mann–Whitney U test) (Figure 2). ROC analysis demonstrated excellent discrimination, with an AUC of 0.989 (95% CI 0.962–1.000) (Figure 3).

At a cut-off of 6.02 Hz, sensitivity was 97.1% (95% CI 85.5–99.5) and specificity 100% (95% CI 72.2–100), with a PPV of 100%, an NPV of 90.9% and an overall accuracy of 97.8% in the present sample. No control subject had a median CBF below 7.1 Hz, so any cut-off between 5.7 and 7.1 Hz produced the same classification and the same maximal Youden index (J = 0.971), indicating that the threshold is stable within this range. Bootstrap resampling of subjects gave a 95% confidence interval of 5.6–7.0 Hz for the optimal cut-off.

A cut-off of 9.4 Hz was required before all patients were classified as abnormal (sensitivity 100%, 95% CI 90.1–100), at which point specificity fell to 60.0% (95% CI 31.3–83.2). Ciliary beat frequency in ALI culture therefore discriminates PCD reliably when it is reduced, but a threshold high enough to capture every patient would misclassify the majority of healthy controls. Normal beating frequency was observed in two patients who exhibited an extremely stiff beating pattern due to reduced ciliary bending, which is also common in PCD; such patients account for the false negatives at every cut-off below 9.4 Hz. Beat frequency alone therefore cannot be used to exclude the diagnosis and must be interpreted together with the beating pattern, ultrastructure and genotype.

According to the Mann–Whitney U test, the difference in CBF between children and adults with PCD was not statistically significant; ciliary beat frequency did not depend on age in patients (Figure 2D).

Thus, our results from the HSVM analysis of ALI-cultured ciliated epithelial samples indicate that this technique is a robust tool for discriminating between patients with PCD and healthy donors, exhibiting high sensitivity (91%) and specificity (89%). This underscores the critical role of ALI cultures in the diagnostic workflow for PCD. However, TEM and the molecular genetic approach remain the primary methods for diagnosis.

**Figure 4:**
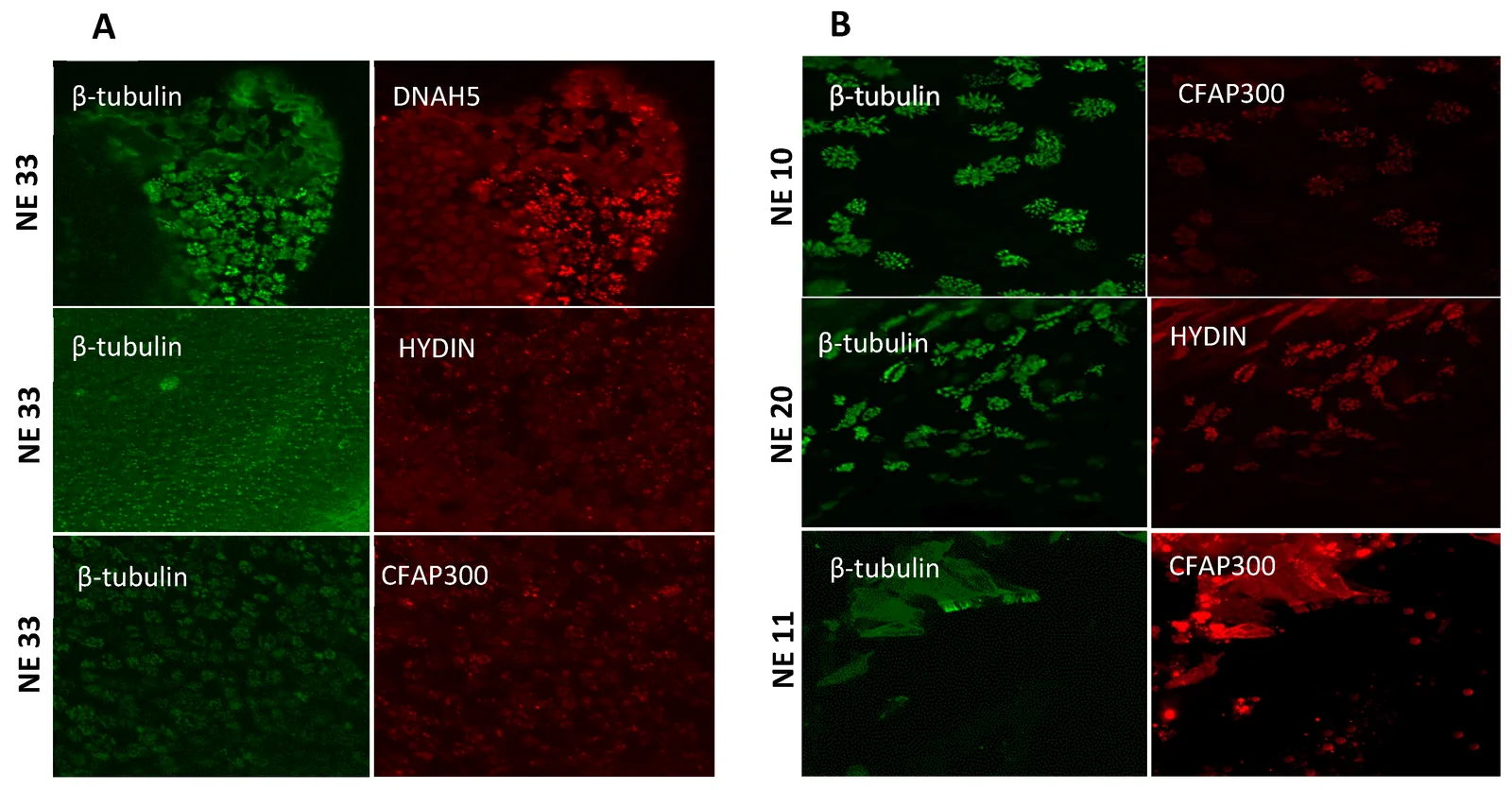
Fluorescent images of cilia in ALI cultures (**A**) from HC and (**B**) from patients with PCD. Magnification 63 times. NE—nasal epithelium, ALI—air–liquid interface, HC—healthy controls, PCD—primary ciliary dyskinesia. β-tubulin—marker of ciliary cells; DNAH5, HYDIN, CFAP300—protein markers of ultrastructural defects.

The fluorescent images of the results of immunofluorescent staining of the ALI culture of ciliary cells on the 24th day of ciliary differentiation from HC and PCD patients with variants in genes *DNAH5*, *DNAH11*, *HYDIN*, *CFAP300* are shown in Figure 4. Compared with healthy controls, ALI-cultured ciliated cells from PCD patients carrying pathogenic variants in *DNAH5*, *DNAH11*, *HYDIN* and *CFAP300* showed markedly reduced or absent axonemal fluorescence signals for the corresponding protein, consistent with loss or mislocalization of the affected structural component of the cilium in patients harboring variants in the relevant gene.

## 4. Discussion

Primary ciliary dyskinesia remains a diagnostically difficult heterogeneous disease. TEM is used to assess the ultrastructural defects in cilia. Immunofluorescence (IF) can demonstrate the absence or mislocalization of ciliary proteins, which is particularly helpful in cases where there are no TEM abnormalities. In individuals with clinical features of PCD, who have not been found to have pathogenic variants of genes, the presence of class 1 defects according to TEM is sufficient to confirm a diagnosis of PCD. The European Commission on PCD compared the accuracy of HSVM, IF, and nitric oxide (NO) levels in exhaled nasal air with a reference, TEM test and/or molecular genetic analysis. The Commission strongly recommended using HSVM, IF and nNO as complementary tests to the TEM and/or molecular genetic methods for PCD diagnostics. However, no additional test is suitable as a stand-alone test for PCD diagnosis and no additional or reference test is suitable for excluding PCD [21,24].

However, although labor intensive, CBF assessment in cultured ciliated cells can help to exclude any secondary defects caused by inflammation or infection of the nasal epithelium in patients [13,25]. Given the complexity and labor-intensive process of ciliogenesis of the ciliated epithelium, CBF assessment is most often performed in ex vivo ciliary motility, which is more sensitive to variations with time and temperature [26].

In a German group of 10 adult healthy volunteers, the CBF range was determined as being from 4.3 Hz to 11.6 Hz (ex vivo) [27]. Another study determined the mean CBF ex vivo for HC (9.3 Hz) and patients with PCD (2.4 Hz) [28].

According to data in the literature, beating of ALI cultures ranged from 6.1 to 9.1 Hz (average of 7.5 Hz) for HCl in the PCD groups, CBF ranged from 0 to 14.3 Hz (average of 5.2 Hz). Hyperkinetic beating was reported in this study, which is why the range is so high. The hyperkinetic beating of cilia was not recorded in our study [29]. Another important means of improving the efficiency of PCD diagnostic workflows concerns the progressive automation of HSVM and IF image analysis. Seminal work by Quinn et al. demonstrated that ciliary motion can be decomposed into quantitative elemental components using a computer vision and machine learning pipeline, achieving automated discrimination of normal versus abnormal ciliary motion in nasal biopsies from patients with PCD, congenital heart disease and heterotaxy [30]. Subsequently, Smith et al. introduced ciliaFA, an ImageJ-based, FFT-driven open-source tool that enables automated, high-throughput measurement of ciliary beat frequency (range 3–52 Hz) and substantially reduces operator-dependent selection bias [31]. Sampaio and colleagues later validated the semi-automated software CiliarMove, which integrates fast Fourier-transform-based CBF quantification with heat-map visualization of ciliary activity; it was shown to discriminate PCD from non-PCD samples in 78% of cases when used as a standalone CBF estimator [32]. More recently, Dimauro et al. developed DeepCilia, a YOLOv8-based deep-learning engine that autonomously detects ciliated strips and estimates CBF with a root mean square error below 1.5 Hz, even from low-frame-rate videos [33]. Beyond motion analysis, Sears et al. established a Gaussian-function-based quantitative modelling framework of human airway ciliary dynamics, providing a reference standard against which automated tools can be benchmarked [34]. For IF microscopy, Jackson and colleagues demonstrated that automated image analysis can reliably discriminate the presence from the absence of ciliary proteins in nasal epithelial cells, supporting its integration into diagnostic pipelines Importantly, a recent ERS BEAT-PCD Clinical Research Collaboration systematic review (Kouis et al.) synthesized 28 PCD-relevant studies on AI-driven and digital-automated tools, concluding that such approaches are technically feasible and could reduce time-to-diagnosis, but that current evidence is largely restricted to single-center, retrospective and small-sample studies [35]. Together, these developments suggest that a fully integrated, AI-assisted pipeline—combining automated CBF/CBP quantification, deep-learning-based TEM axonemal defect classification and automated IF pattern recognition—is an attainable near-term goal and could substantially reduce the workload of expert operators while improving inter-center reproducibility.

Although ALI cultures represent a more relevant model, they require considerable time and resources to establish. The approach, which focuses on direct sampling methods, allows for immediate analysis and provides a balance between clinical efficacy and CBF accuracy. Future research is needed, including ALI-culture assessments, to complement and expand our results, further enriching the understanding of the cilia function in PCD [36].

ALI cultures are also used as models for testing therapeutic drug delivery and microbial infection, e.g., the effect of drugs on ciliary activity, and airway barrier function during bacterial infection [37,38].

The results are consistent with existing research and demonstrate the need for comprehensive diagnostic approaches. The ciliogenesis of the cilia epithelium and CBF assessment with software can significantly improve the diagnosis of PCD. This study highlights the need for multiple methods to provide a definitive diagnosis.

**The strengths and the limitations of this study.**

The main strength of this study is that the diagnosis of PCD was established independently of the test in every participant: all 35 patients had a molecular genetic diagnosis, and the cohort was additionally characterized using the transmission electron microscopy and immunofluorescence staining of ALI cultures. Functional assessment was therefore compared against a composite reference standard rather than against clinical suspicion alone. The use of ALI-cultured cells rather than ex vivo samples reduces the contribution of secondary ciliary dyskinesia caused by inflammation or recent infection, which is a recognized source of misclassification in ex vivo analysis. CBF was quantified using dedicated validated software rather than by manual counting, which removes inter-observer variability from the frequency estimate. The cohort includes both children and adults, and, to our knowledge, represents the largest series of genetically confirmed PCD patients studied by HSVM in ALI culture reported from the Russian Federation. Several limitations should be acknowledged. First, this is a single-center study, and the cut-off derived here reflects the acquisition conditions, culture protocol and analysis software of one laboratory; multicenter validation is required before the threshold can be transferred to other settings. Second, although the patient cohort is comparatively large for a rare disease, the control group comprised only 10 healthy donors, which yields wide confidence intervals for specificity. Third, the study has a case–control design, in which the proportion of PCD patients is far higher than in any population in which the test would be applied; consequently, predictive values derived directly from this sample are not transferable to clinical practice and must be re-estimated for the relevant pre-test probability. Fourth, ALI-cultured and ex vivo materials were not compared directly in the same subjects, so the superiority of ALI culture asserted here rests on the absence of secondary dyskinesia rather than on a paired within-subject comparison; such a comparison is the subject of ongoing work. Fifth, the analysis was restricted to beat frequency and did not include quantitative assessment of beat pattern or amplitude, which explains why two patients with a stiff beating pattern were not identified by the frequency threshold; this limits the use of CBF as a stand-alone screening parameter. Sixth, nasal nitric oxide measurement, which is recommended as a complementary test, was not available for this cohort. Seventh, healthy controls were not age-matched to patients, although no dependence of CBF on age was detected within the patient group. Finally, the number of patients per genotype was too small to allow for an analysis of genotype–function correlations, and CBF was assessed at a single time point of differentiation (day 24), so the stability of the measure across culture time was not evaluated.

## 5. Conclusions

Analyzing CBF in ALI cultures is more accurate than the ex vivo method. Moreover, the creation of databases of ALI cultures, combined with genotypic and structural abnormalities, is necessary both for diagnosis and, in the future, for use as models in the development of targeted therapies and in assessing their effectiveness.

## Figures and Tables

**Table 1:** Clinical and anamnestic data, instrumental findings of patients with PCD.

№	Gene	Variants		Adult/Child	Gender M/F	CRSwNP/CRSsNP	Hearing Loss (Degree)	Bronchiectasis	Situs Inversus	TEM (Class)	Seeding of *P. aeruginosa*
1.	*DNAAF11* *homozygous mutation*	c.436G>C	p.Asp146His	A	F	+	-	+	+	1	+
2.	*DNAAF11* *homozygous mutation*	c.436G>C	p.Asp146His	A	F	+	-	+	-	1	+
3.	*DNAAF11*	c.436G>C	p.Asp146His	C	M	+	-	-	-	-	**-**
		c.1011A>G	p.Gln337Gln								
4.	*CFAP300* *homozygous mutation*	c.198_200delinsCC	p.(Phe67ProfsTer10)	A	M	+	-	+	+	1	+
5.	*CFAP300* *homozygous mutation*	c.198_200delinsCC	p.(Phe67ProfsTer10)	A	F	+	1	+	+	1	+
6.	*CFAP300* *homozygous mutation*	c.198_200delinsCC	p.(Phe67ProfsTer10)	A	F	+	-	+	-	1	-
7.	*HYDIN* *homozygous mutation*	c.1443T>G	p.(Tyr481Ter)	C	M	+	-	+	-	-	-
8.	*HYDIN*	c.12899G>C	p.(Cys4300Ser)	C	M	+	-	+	+	2	+
		c.3291A>G	p.(Ile1097Met)								
9.	*HYDIN*	c.13989delC	p.(Ile4664Serfs*33)	C	F	+	-	-	-	-	+
		c.7483G>A	p.(Asp2495Asn)								
10.	*HYDIN*	c.13989delC	p.(Ile4664Serfs*33)	C	F	+	-	-	-	-	+
		c.8791C>T	p.Arg2931Cys								
11.	*DNAH14*	c.9685C>T	p.Arg3229Ter	C	F	+	-	-	+	1	**-**
		c.13639-2del	splicing?								
12.	*FSIP2*	c.12793G>T	p.Gly4265Term	A	F	+	3–4	**+**	**-**	-	**+**
		c.16671A>C	p.Glu5557Asp								
13.	*DNAAF4*	c.988C>T	p.Arg330Trp	C	M	+	1	+	+	-	-
		c.583del	p.Ile195Ter								
14.	*DNAH17*	c.12583_12585del	p.(Glu4195del)	A	M	+	-	-	-	2	-
		c.10202C>T	p.(Pro3401Leu)								
15.	*DNAH11*	c.2966G>A	p.R989Q	C	M	+	-	-	-	N	-
		c.7294C>T	p.R2432W								
16.	*DNAH11*	c.4360_4361delinsTT	p.E1454L	C	M	+	-	-	-	-	+
		c.8501T>C	p.M2834T								
17.	*DNAH11*	c.13140del	p.(Phe4380LeufsTer7)	C	F	+	1	+	-	2	-
		c.10751A>C	p.(His3584Pro)								
18.	*DNAH11*	c.6983+1G>A	p?	A	F	+	-	+	+	1	-
		c.5461-10A>G	p?								
19.	*DNAH11*	c.1231G>T	p.Gly411*	C	M	+	1	-	+	1	-
		c.13373C>T	p.Pro4458Leu								
20.	*DNAH11*	c.13140del	p.(Phe4380LeufsTer7)	C	F	+	-	-	-	2	+
		c.10751A>C	p.(His3584Pro)								
21.	*DNAH9*	c.2080A>G	p.Ile694Val	A	F	+	-	+	-	N	-
		c.3593G>C	p.Trp1198Ser								
22.	*DNAH5*	c.10815delT	p.(Pro3606HisfsTer23)	C	F	+	-	+	-	1	-
		c.10556-1G>A	splice								
23.	*DNAH5*	c.10815delT	p.(Pro3606fs)	A	F	+	1	+	+	1	+
		c.3074dupC	p.(Ala1026fs)								
24.	*DNAH5*	c.10815del	p.(Pro3606HisfsTer23)	A	M	+	-	+	+	1	-
		c.8390T>G	p.(Leu2797Arg)								
25.	*DNAH5*	c.5177T>C	p.Leu1726Pro	C	F	+	-	+	-	1	-
		c.7596T>A	p.Tyr2532Ter								
26.	*DNAH5*	c.11653C>T	p.(Arg3885*)	C	M	+	-	-	+	1	-
		c.13604_13609de	p.(Val4535_Tyr4536del								
27.	*DNAH5*	c.8440_8447del	p.(Glu2814*)	A	F	+	-	+	-	1	+
		c.13369T>C	p.(Trp4457Arg)								
28.	*DNAH5*	c.8403G>A	p.Trp2801	A	M	+	-	-	+	1	-
		c.8390T>G	p.(Leu2797Arg)								
29.	*OFD1*	c.2674C>T	Gln892X	C	M	+	-	+	-	2	-
30.	*OFD1*	c.2674C>T	Gln892X	A	M	+	1–2	+	-	1	+
31.	*DNAL1* *homozygous mutation*	c.23_24del	p.Lys8Argfs*16	C	M	+	-	-	-	1	-
32.	*CCDC39* *homozygous mutation*	c.2492_2496del	p.(Met831ThrfsTer7)	A	F	+	-	+	+	2	+
33.	*CCDC40* *homozygous mutation*	c.2440C>T	p.(Arg814Ter)	A	F	+	2	+	-	2	+
34.	*CCDC103* *homozygous mutation*	c.461A>C	p.His154Pro	C	M	+	-	-	-	1	+
35.	*DNAAF1*	c.655T>C	p.(Cys219Arg)	A	M	+	1	+	+	1	+
		c.1384C>T	p.(Gln462Ter)								

Abbreviations: A—adult, C—child; F—female; M—male, N—normal. TEM—transmission electron microscopy.

## Data Availability

The data presented in this study are available on request from the corresponding author due to legislation on data transfer.
